# Illegal fishing with electrofishing devices in the Po river basin, Emilia Romagna, Italy

**DOI:** 10.1038/s41598-021-93015-z

**Published:** 2021-07-27

**Authors:** Sandro Mazzariol, Giorgia Corazzola, Silva Rubini, Francesco Quaglio, Alberto Perolo, Andrea Gustinelli, Marialetizia Fioravanti, Chiara Anna Garbarino, Maria Cristina Fontana, Paolo Frisoni, Rosa Maria Gaudio, Cinzia Centelleghe

**Affiliations:** 1 Department of Comparative Biomedicine and Food Science, University of Padua, Legnaro, Italy; 2 Experimental Zooprophylactic Institute of Lombardy and Emilia Romagna, Brescia, Italy; 3 Department of Veterinary Medical Sciences, Alma Mater Studiorum University of Bologna, Bologna, Italy; 4 Department of Morphology, Surgery and Experimental Medicine, Section of Legal Medicine, University of Ferrara, Ferrara, Italy; 5 Department of Medical Sciences, University of Ferrara, Ferrara, Italy

**Keywords:** Pathogenesis, Ichthyology

## Abstract

Electric fishing is an illegal hunting method, unfortunately widely used by poachers to paralyze fish and to catch many animals in a short time. In Italy, it is authorized only for scientific and conservative purposes. Between 2014 and 2018, the Ferrara section of the Experimental Zooprophylactic Institute of Lombardy and Emilia Romagna, Italy, received nine cases of potentially illegal electric fishing in Po river and its tributary rivers. Necropsies were performed following standard protocols and samples of different tissues were collected and examined using histochemical and immunohistochemical techniques. Gross lesions frequently observed were circulatory alteration phenomena (i.e. multi-organ hyperemia, hemorrhages and congestion, hemopericardium), also found histologically, in addition to multifocal degenerative and necrotic muscular processes that could be attributed to injuries from electric current, as already reported in literature. Immunohistochemical investigations confirmed degenerative and necrotic lesions with myoglobin depletion and a corresponding fibrinogen accumulation. Myoglobin globules were also detected in the renal parenchyma, as consequent of rhabdomyolysis. The results of this study allowed to correlate electric fishing to gross, histologic and immunohistochemical lesions, which together constitute a pathognomonic picture to be considered a reference standard in this type of illegal controversy.

## Introduction

Electrical fishing (or electrofishing) includes different methods for harvesting that have in common the application of an electric field to paralyze fish into a water basin; this method allows to catch many animals in less time respect other traditional fishing techniques^1–3^. The characteristic behavior and immobilization of fish are supposed to be the results of the electrical field that stimulates a muscular reaction, either involving the central and peripheral nervous system or not.

It is not yet completely clear the mechanism that leads to immobilization in fishes. Two theories have been described to explain this phenomenon, both with undoubted elements of truth: on one side, the theory named “Biarritz Paradigm” considers immobility a reaction to the electrostimulation of both the nervous system (central and autonomic) and muscular tissue (i.e. a reflex response)^1,4^. The other hypothesis, the so called “Bozeman Paradigm”, states that electrical stimulation only affects the central nervous system (CNS) and when it is overwhelmed, the fish response is epileptic seizures^1^. The effectiveness of the electric field depends on many factors including the type of electrical waveform, the concentration of ions into the water and relative conductivity, the water temperature, the distance between fish and electrodes, but also the involved species and the size of each single animal^5^.

The Italian legal framework prohibits electrofishing for commercial use and in sport fishing. It is allowed only for scientific purposes, such as to study fish populations in small water basins, where no other approaches are available, to collect fish from channels during the dry season, to regulate excessive populations of unwanted species, or to capture and select breeding animals at spawning sites^6^. Despite laws prohibit this practice, public media and authorities frequently report cases of poachers using this method, suggesting that it is widely used in Italy^6^. Illegal electrofishing seems to be commonly used in rivers with large fish populations, like the Po river catchment basin, as discussed by Gaudenzi^6^. Poachers produce homemade electro-devices, using car batteries with electric cables immersed in water. Electric fields caused by these rudimentary devices are uncontrolled and can affect in its range of action all fishes of all size and age classes, up to species, which do not constitute a commercial interest. During inspections performed by the Authorities, electrofishing equipment is not usually found and so cannot be related to the caught fishes, since it is often thrown away into the water or hidden^6^. Further tools are needed to support in the Court the hypothesis of electrofishing in similar illegal cases. In order to develop a specific forensic approach, animals found in suspected poaching episodes in Northern Italy (Po River) between 2014 and 2018 were retrospectively investigated in this study and compared with a certainly assessed poaching episode occurred in June 2018. Pathological analyses carried out on these field cases were aimed to assess post-mortem evidences related to the exposure to electro-device usage in fish.

## Results

### Gross examination

**Figure 1:**
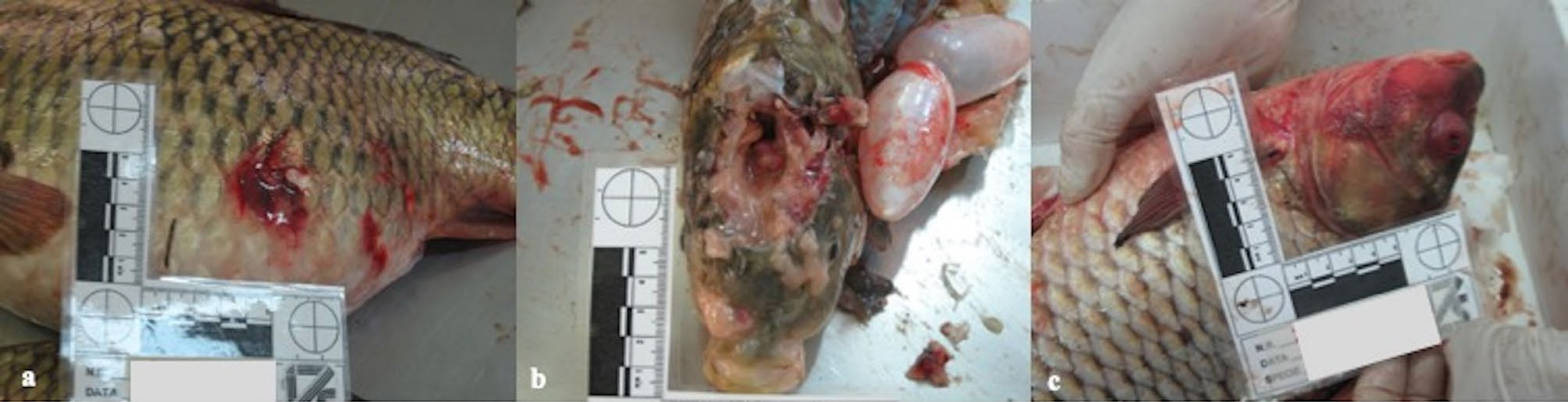
Main gross lesions. (**a**) Multifocal moderate cutaneous hemorrhages, skin of the ventro-lateral part of the body, common carp. (**b**) Diffuse mild to moderate meningeal hyperemia, brain, common carp. (**c**) Focally extensive moderate cutaneous hemorrhages and diffuse hyperemia with severe exophthalmos, European crucian carp.

All six fish belonging to case 1, identified as positive control, presented at the gross examination diffuse moderate to severe hyperemia of skin, mainly in the ventral region and associated with multifocal moderate to severe cutaneous hemorrhages (Fig. 1a), gills and brain (Fig. 1b), various amount of partially digested contents in the stomach, moderate to severe hemopericardium, and multifocal intra-ocular hemorrhages (Fig. 1c).

In the other eight cases (cases 2–9), 121 fish were grossly evaluated and the most commonly reported findings were severe multifocal hyperemic and hemorrhagic findings mainly in the skin, gills and skeletal muscles (presented by the 100% of the animals, 121/121), and moderate to severe hemopericardium (84%, 102/121). Less frequently encountered lesions were bulging of the eyes (44%, 53/121) and multifocal ocular hemorrhages (31%, 38/121). A small number of animals (0.6%, 7/121) had poorly or partially digested food remains into the stomach, as reported for the control case. No lesions associated with the vertebral column were observed.

### Microscopic examination

**Figure 2:**
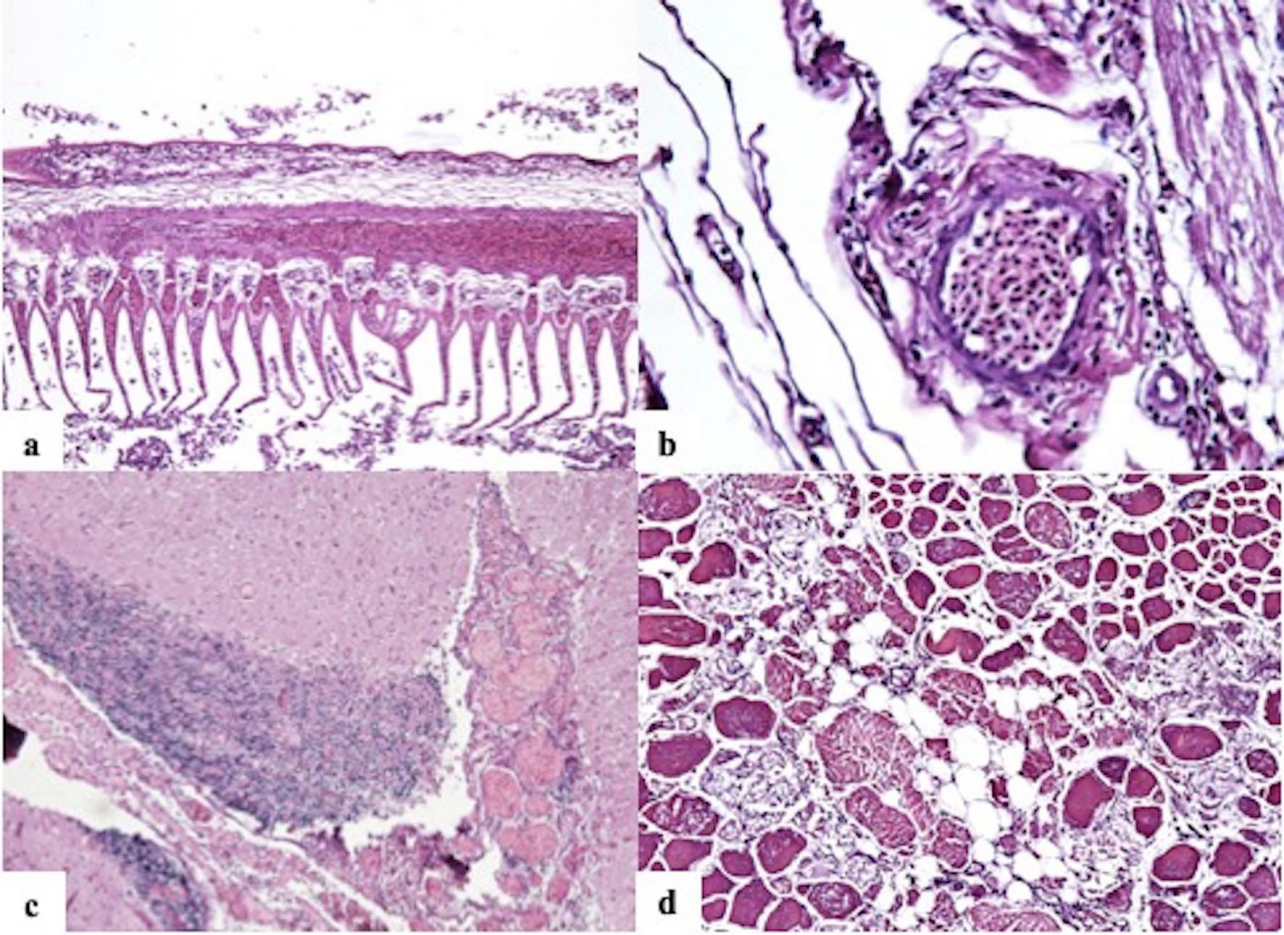
Main histological lesions. (**a**) Moderate to severe diffuse congestion and epithelial sloughing, gills, common carp. Hematoxylin–eosin (HE), ×4 magnification. (**b**) Diffuse severe congestion of cardiac and coronary arteries, heart, common carp. HE, ×40 magnification. (**c**) Diffuse moderate to severe vascular congestion of encephalic vessels and choroid plexus associated with perivascular infiltrate of glial cells, brain, common carp. HE, ×4 magnification. (**d**) Multifocal to coalescing, severe Zenker’s necrosis, skeletal muscle, common carp. In the necrotic areas, loss of cross striations and nuclei, hyaline appearance of the cytoplasm, and fragmented fibers are evident. HE, ×20 magnification.

Five fish of case 1 (four common carps and one European crucian carp) were histologically examined and the most important and diffuse lesions they presented were numerous multifocal foci of extravasated red blood cells (multifocal moderate to severe acute hemorrhages) and multifocal to diffuse moderate to severe congestion in the skin, skeletal muscle, gills (Fig. 2a), heart (Fig. 2b), liver, spleen, kidney (also in the hematopoietic tissue) and brain (Fig. 2c). Multifocally muscular fibers, in the five animals, were swollen, with loss of cross striations and nuclei, and a hyaline appearance, interpreted as Zenker’s necrosis (Fig. 2d). The four common carps had mild gill disease caused by numerous protozoal elements, identified as *Trichodina* spp. Two of these showed multifocal moderate to severe inflammatory infiltrate composed mainly of lymphocytes and granular eosinophilic mast cells, interpreted as multifocal moderate chronic branchitis. The kidney exhibited vacuolar degeneration of the tubular epithelium (3/5) and necrosis of the tubular epithelium (1/5); while liver presented a diffuse mild steatosis (3/5).

In the other 32 fish belonging to cases 2–9 histologically observed, multifocal to coalescing, moderate to severe hemorrhages and hyperemic findings were confirmed in skin, gills and muscle of all individuals. Furthermore, multifocal to coalescing, moderate to severe Zenker’s necrosis of skeletal muscle fibers were also observed in 28/32 animals (87%). Additional common lesions were multi-organ, multifocal to coalescing, moderate to severe congestion and multifocal, moderate hemorrhages (65%, 21/32). Other lesions were detected in gills, such as epithelial mild to moderate multifocal necrosis (28%, 9/32) and epithelial sloughing (28%, 9/32). All macroscopic and histological specific findings showed in each case are summarized in Supplementary Table 1.

### Immunohistochemical (IHC) analysis

**Figure 3:**
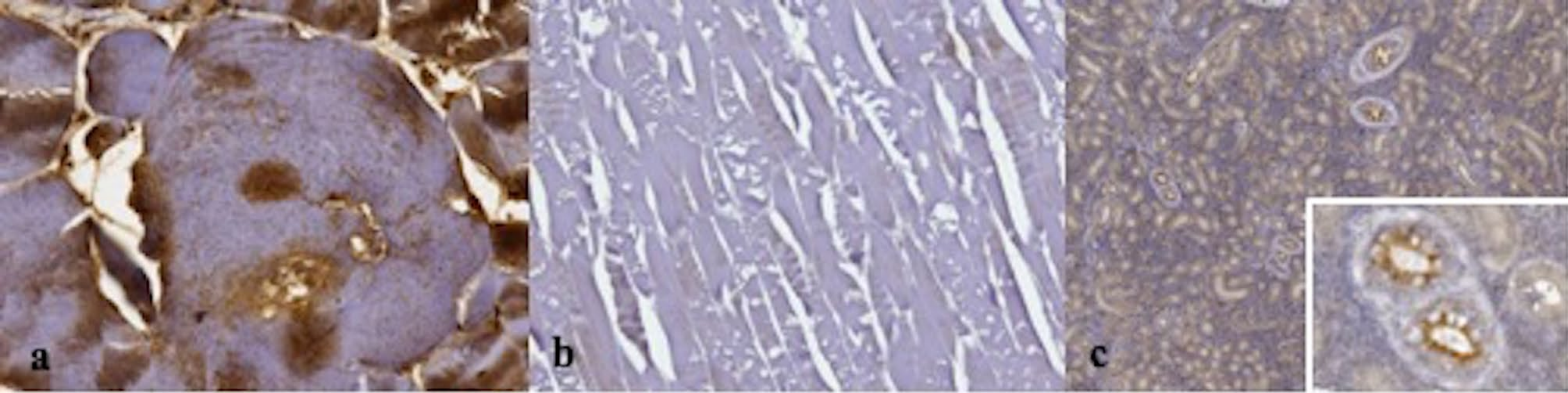
Immunohistochemical (IHC) pattern. (**a**) Strong intracytoplasmic positive immunoreaction for fibrinogen in affected myocytes, skeletal muscle, common carp. IHC for fibrinogen ad Mayer’s hematoxylin counterstain, ×40 magnification. (**b**) Severe diffuse depletion of myoglobin in degenerated myocytes is demonstrated by the absence of immunoreaction for myoglobin, skeletal muscle, common carp. IHC for myoglobin and Mayer’s hematoxylin counterstain, ×10 magnification. (**c**) The apical cytoplasm of tubular cells shows positive immunoreaction for myoglobin, kidney, common carp, ×4 magnification. At the right corner a particular of tubular cells. IHC for myoglobin and Mayer’s hematoxylin counterstain, ×40 magnification.

The IHC confirmed the degenerative/necrotic changes to muscular tissue, since in skeletal muscle fibers were characterized by multifocal, moderate to severe, fibrinogen accumulation and myoglobin depletion (Fig. 3a,b). Furthermore, myoglobin was detected in renal collector tubules (Fig. 3c), confirming muscular damage and rhabdomyolysis.

No evidence of SVCV infections was reported in the common carps belonging to case 2.

## Discussion

The use of electrofishing is prohibited Italy since, if applied constantly, it could result in serious depletion of fish resources. In fact, the electric current affects all species of fish present in the area of application of the electric field, making electrofishing an unspecific way of catching aquatic animals. Moreover, electrical fishing could be dangerous for fishermen depending on electric current voltage, causing ventricular fibrillation, asystolia, till cardiac arrest and death, even more if the electrical device used is “homemade” and then unsafe^7–9^. As stated above, Authorities are not usually able to find poachers with the captured fish and the electro-devices simultaneously, as occurred in case 1 in this study and, for this reason, a better understanding of post-mortem evidences associated with this illegal type of fishing could support the investigation needed for legal actions.

In humans and animals, death for electrocution can determine electrothermal burns in high-voltage accidents or in low-voltage ones with a prolonged contact with an electric device^10^. Internal injuries are rare in low-voltage accidents: only signs of acute circulatory failure like acute venous congestion of shock organs with hemorrhagic changes in the trachea, heart, and lungs, are the main gross findings during necropsy^10^. At the histological examinations, tissue injuries can vary depending on voltage and contact durations. The distribution can also differ depending on the pathway of the current. Edema, degeneration and necrosis of muscle cells can develop after structural damage to the cell membranes. Complications as rhabdomyolysis and myoglobinuria or hemolysis and hemoglobinuria can occur in survivors, resulting in renal failure. In fact, electricity tends to flow along vessels since blood is a good conductor, causing damage to endothelial cells and myocytes and subsequent thrombosis, at any time after the accident^11^.

In fish, die-off due to electrocution were reported after lighting strikes in water or stray voltage for electrofishing equipment’s malfunction. Electricity could cause severe muscular hypercontraction with subsequent extensive muscular hemorrhages, myonecrosis and possible spinal fractures^12–14^. Previously, Snyder and colleagues described also gross hemorrhages of tissues of internal organs associated to electroshocking in fishes^15^. No pathological changes on the vertebral column were detected, presumably because the voltage was variable and not high enough to cause a muscular contraction leading the rupture of the column^6^.

Both in case 1 and in the other eight cases herein investigated, skin, gills, eyes and skeletal muscles presented congestion and hemorrhages confirmed also during histological examination. Furthermore, multifocal areas of flaking and disepithelization associated with erosions and hemorrhages characterized histologically the skin, likely caused by excessive intensity of the electric fields. In addition to the hemorrhagic lesions of gills, necrosis and cellular sloughing were reported^16,17^. Congestive/hemorrhagic lesions of gills, heart and skin and acute muscular Zenker’s necrosis, without inflammatory responses and evidence of other transmissible pathogens, are consistent with acute injuries caused by electric current in fish. Due to rapid post-mortem changes in fish, accelerated by electrocution, IHC was used to support evidence of muscular damage and acute rhabdomyolysis. Myoglobin and fibrinogen detection through IHC reaction was already used in other species to support ante-mortem damages: sarcoplasmic myoglobin depletion coupled to sarcoplasmic fibrinogen deposition support the diagnosis of acute degenerative muscle lesions^18,19^. As summarized in Supplementary Table 1, the above reported lesions were not related to the species.

Death by electrocution remains difficult to diagnose also in humans, if external electric marks are not present, in particular under field conditions as those included in this investigation. Furthermore, biochemical analyses and scanning electronic microscope protocols, used in human forensic medicine as a contribution to the diagnosis, are not yet implemented and standardized for wildlife species, as fish^20^. Experimental studies confirm that the severity of related damages is associated with different features of the device used^21^, but in the case here presented these characteristics were not detected during seizing by the Authorities and cannot be compared with known examples in the existing literature.

All the above-mentioned pathological changes are consistent with a non-specific sub-acute degenerative and necrotizing cardiac and skeletal myopathy with subsequent rhabdomyolysis. These non-specific lesions can be diagnosed as a consequence of an electrocution only when the exposure to an electric source can be clearly proven, as occur for raptors died touching power lines and human fatalities^22,23^. In fact, only the spatial and temporal association between muscular damage with the hemoglobinuric nephrosis and the concurrent use of an electric device observed in case 1 helped to point out electrocution as the likely cause of death^22,23^, even if the limited number of animals belonging to this group impaired statistical analyses. This casualty along with the absence of additional pathological changes assessed with post-mortem investigations and the absence of specificity of the reported changes, illegal electrofishing has also been hypothesized in all other eight cases where circumstances were highly suggestive. It should be noted that this study was conducted retrospectively on field cases submitted for diagnostics in a period of 5 years and a standardized data and samples collection protocols was not implemented a priori as it was not possible to plan the size of the control group, not being an experimental condition. Furthermore, in similar cases, additional ancillary examinations, including microbiological and toxicological studies, should be performed to exclude any other differential diagnosis. These procedures should also be carried out on all the examined animals. Unfortunately, considering the high number of animals and due to economical restrains and poor scientific interests, other diagnostic tools such as those related to cardiac damage cannot be implemented in this study, even if of certain interest in other aquatic species affected by the same problem, as freshwater dolphins^23^.

Besides the limitations of this study, our efforts were aimed to support the local Authorities in countering illegal fishing and the use of electrofishing for forbidden purposes, offering a valuable diagnostic tool. The spatial and temporal use of a functioning electric device contemporary to the presence of dead fish in case 1 is a casualty that offers a unique opportunity to enforce the research on this problem affecting freshwaters in Northern Italy. The reported muscular and renal findings have increased the knowledge on death due to electroshock in fish stressing the mechanism of death in these cases and identifying the cause of death. The above microscopic findings underline the relevance to include a standardized sampling procedures of muscular and renal tissues in suspected poaching cases to confirm the mechanism of death. All the collected information is a valuable contribution to the emergent field of the veterinary forensic pathology but also offers a valuable approach in the subsequent investigation underlying the need of a common and standardized approach in similar cases.

## Methods

### Sample collection and post-mortem examination

Between 2014 and 2018 the laboratory of pathology of the Experimental Zooprophylactic Institute of Lombardy and Emilia Romagna, Italy (IZSLER) in Ferrara received nine cases (127 fish) of potentially illegal electrical fishing. Data of fish are reported in Supplementary Table 1. The eight species examined included common carp (*Cyprinus carpio*, N = 68), freshwater bream (*Abramis brama*, N = 30), wels catfish (*Silurus glanis*, N = 11), pike-perch (*Sander lucioperca*, N = 9), European crucian carp (*Carassius carassius*, N = 2), flathead grey mullet (*Mugil cephalus,* N = 3), silver carp (*Hypophthalmichthys molitrix*, N = 3), lagermouth black bass (*Micropterus salmoides*, N = 1).

All the fish were seized to poachers by the Authorities during the night along Po river and its tributary rivers. Only in one case occurred in June 2018, named case 1, fish (four common carps and two European crucian carps) were confiscated to poachers while they were using an electrofishing device: for these reasons, this group was considered a reference case and positive control. In the other cases, the electrofishing device was not found and illegal poaching was only suspected. The electrofishing device was composed by four car batteries (> 95 Ah) connected with cables to a 12 V voltage regulator and two cables directly into the water. In all other 8 events, poachers were found with dead prey without any tool for the illegal capture.

After death, animals were maintained refrigerated (5 °C) and necropsies were performed for 127 animals within 2–4 h after death according to a standardized protocol^24^. Samples of gills, skin, skeletal muscle, brain, eyes, heart, swim bladder, liver, spleen, pancreas, gonad and kidney were collected in 37 fish, depending on gross findings during post-mortem examination and on the conservation status of the animal, and were evaluated with histochemical and IHC techniques. Fishes belonging to case 2 were catch during the warm seasons and to exclude an infection by the Spring Viremia of Carp virus (SVCV), responsible for hemorrhagic post-mortem findings in this period^25^, virological examination was carried out using cell culture and viral extraction on five animals according to OIE Manual of diagnostic tests for aquatic animals.

### Histochemical and immunohistochemical (IHC) analysis

Collected samples were fixed in 10% neutral-buffered formalin for more than 72 h and subsequently paraffin-embedded. Four micrometer (µm) sections were prepared and stained with hematoxylin and eosin (H&E) for routine microscopic examination.

Skeletal muscle and kidney from the common carps belonging to case 1 (the positive control) were also examined using IHC techniques, to assess muscular damages. In detail, muscular degeneration/necrosis were investigated by IHC evaluating possible accumulation of fibrinogen, suggesting an *intra-vitam* damage, and the contemporary myoglobin depletion in muscle fibers. Furthermore, evidence of myoglobin in renal tissues supports the hypothesis, being an indication of myoglobinuria due to muscular damage^18^. Used primary antibodies were respectively anti-human rabbit polyclonal fibrinogen and rabbit polyclonal myoglobin (Dako, Santa Clara, California). In the investigated cases, four µm sections of muscular and renal tissues were mounted on Superfrost Plus microscope slides (Menzel GmbH, Braunschweig, Germany) and dried at 37 °C for 30 min. IHC staining was performed using an automated immunostainer (BenchMark, VentanaMedical Systems Inc., Tucson, Arizona) that included dewaxing and rehydration, heat-induced antigen retrieval (30 min at 95 °C), primary antibody incubation, antigen detection with ultraView Universal DAB kit (Ventana Medical Systems) and counterstaining with hematoxylin. Finally, slides were manually dehydrated through a graded series of alcohols and mounted (Eukitt mounting medium; Electron Microscopic Services, Fort Washington, Pennsylvania). Primary antibody dilutions were performed using a commercial antibody diluent (Ventana Medical Systems). Specific antibodies feature, dilution, time and temperature of incubation are specified in Table 1.

**Table 1:** Characteristics of antibodies used for IHC analysis.

Antibody (Ab)	Features	Dilution	Incubation of primary Ab—temperature (°C) and time (min)	Localization
Fibrinogen	Dako; Polyclonal rabbit anti-human	1:50	37 °C for 32 min	Intra-cytoplasmatic
Myoglobin	Dako; Polyclonal rabbit anti-human	1:200	37 °C for 28 min	Intra-cytoplasmatic

## Supplementary Information

Supplementary Table 1.

## References

[1] Beaumont WRC (2016). Electricity in Fish Research and Management: Theory and Practice.

[2] Hartley WG (1980). The use of three-phase current for electrical fishing. Fish. Manag..

[3] Sharber NG, Carothers SW, Sharber JP, De Vos JC, House DA (1994). Reducing electrofishing-induced injury of rainbow trout. N. Am. J. Fish. Manag..

[4] Sharber NG, Black JS (1999). Epilepsy as a unifying principle in electrofishing theory: A proposal. Trans. Am. Fish. Soc..

[5] Beaumont WRC, Taylor AAL, Lee MJ, Welton JS (2002). Guidelines for Electric Fishing Best Practice. R&D Technical Report W2–054/TR.

[6] Gaudenzi D (2016). Manuale di antibracconaggio per guardie ittiche volontarie dell’Emilia Romagna.

[7] Balachandran A, Krishnan B, John L (2013). Accidental deaths due to electrocution during electro-fishing. J. Evol. Med. Dent. Sci..

[8] Fodor L, Bota IO, Abbas Y, Fodor M, Ciuce C (2011). Electricity and fishing—A dangerous mix. Burns.

[9] Lee, E. The physiological effects of electrofishing. *California-Nevada Wildlife Transactions.* 59–72 (1984).

[10] Schulze C, Peters M, Baumgartner W, Wohlsein P (2016). Electrical injuries in animals: Causes, pathogenesis, and morphological findings. Vet. Pathol..

[11] Price TG, Cooper MA, Marx J, Walls R, Hockberger R (2013). Electrical and lightning injuries. Rosen’s Emergency Medicine—Concepts and Clinical Practice.

[12] Pasnik DJ, Smith SA, Wolf JC (2003). Accidental electroshock of fish in a recirculation facility. Veterinary Record.

[13] Roberts, R. J. Miscellaneous non-infectious disease. In: *Fish Pathology*, 4th edn, 425–438 (2012).

[14] Turnbull, J. Musculoskeletal system. In: *Systemic Pathology of Fish*, 2nd edn, 289–311 (2006).

[15] Snyder DE (2003). Electrofishing and Its Harmful Effects on Fish.

[16] Hauck FR (1949). Some harmful effects of the electric shocker on large rainbow trout. Trans. Am. Fish. Soc..

[17] Taylor GNL, Cole S, Sigler WF (1957). Galvanotaxic response of fish to pulsating direct current. J. Wildl. Manage..

[18] Brancaccio P, Lippi G, Maffulli N (2010). Biochemical markers of muscular damage. Clin. Chem. Lab. Med..

[19] Sierra E, de Los E, Monteros A, Fernández A, Díaz-Delgado J, Suárez-Santana C, Arbelo M, Sierra MA, Herráez P (2017). Muscle pathology in free-ranging stranded cetaceans. Vet. Pathol..

[20] Cooke SJ, Bunt CM, McKinley RS (1998). Injury and short term mortality of benthic stream fishes—A comparison of collection techniques. Hydrobiologia.

[21] Mondello C, Micali A, Cardia L, Argo A, Zerbo S, Spagnolo EV (2018). Forensic tools for the diagnosis of electrocution death: Case study and literature review. Med. Leg. J..

[22] Kagan RA (2016). Electrocution of raptors on power lines: A review of necropsy methods and findings. Vet. Pathol..

[23] Thomas PO, Gulland FMD, Reeves RR, Kreb D (2019). Electrofishing as a potential threat to freshwater cetaceans. Endang. Species Res..

[24] Yanong, R. P. E. Necropsy techniques for fish. *Seminars in avian and exotic pet medicine*. **12**(2), 89–105 (2003).

[25] World Organisation for Animal Health. *Manual of Diagnostic Tests for Aquatic Animals*. Chapter 2.3.9. (2019)

