# Supplementary material for: Illegal fishing with electrofishing devices in the Po river basin, Emilia Romagna, Italy
Source: Sci Rep. 2021 Jul 27;11:15270. doi: 10.1038/s41598-021-93015-z (PMC8316363; doi:10.1038/s41598-021-93015-z)
Supplement: Supplementary file 1 — Supplementary Table 1. [file 41598_2021_93015_MOESM1_ESM.pdf]

Illegal fishing with electro-fishing devices in the Po river basin, Emilia Romagna, Italy

Sandro Mazzariol[1], Giorgia Corazzola[1,*], Silva Rubini[2], Francesco Quaglio[1], Alberto Perolo[1], Andrea Gustinelli[3], Marialetizia Fioravanti[3], Chiara Anna Garbarino[2], Maria Cristina Fontana[2], Paolo Frisoni[4], Rosa Maria Gaudio[5], Cinzia Centelleghe[1].

1: Department of Comparative Biomedicine and Food Science, University of Padua, Padua, Italy;

2: Istituto Zooprofilattico Sperimentale della Lombardia e dell'Emilia Romagna (IZSLER), Brescia, Italy;

3: Department of Veterinary Medical Sciences, Alma Mater Studiorum University of Bologna, Bologna, Italy;

4: Department of Morphology, Surgery and Experimental Medicine, Section of Legal Medicine, University of Ferrara, Ferrara, Italy;

5: Department of Medical Sciences, University of Ferrara, Ferrara, Italy.

*: Corresponding author: Giorgia Corazzola, DVM

giorgia.corazzola@gmail.com

Viale dell'Università 16, 35020 Legnaro (PD)

0039049.8272963

Supplementary Table 1. Macroscopic and microscopic lesions find in the analysed fish.

The table includes the number and the species of fish included in each case, and it describes gross and histological findings of all the fish examined.

Case number	No. of animals	Species (No. of animals)	Macroscopic lesions	Microscopic lesions	Suspected cause of death
1	6	*Cyprinus carpio (4)*	**Skin**: diffuse moderate hyperemia, mainly in the ventral region (4/4), multifocal moderate hemorrhages (4/4);<br>**Eyes**: multifocal intraocular hemorrhages;<br>**Gills**: diffuse severe hyperemia (4/4);<br>**Heart**: hemopericardium (4/4);<br>**Stomach**: partially digested gastric content (4/4).	*4 animals examined*<br>**Skin**: multifocal moderate to severe hemorrhages (3/4);<br>**Gills**: mild gills disease associated to Trichodina spp. protozoal elements (4/4), multifocal severe inflammatory infiltrate composed by lymphocytes, granular eosinophilic mast cells with rare bacteria aggregates (2/4), multifocal moderate epithelial necrosis and sloughing (3/4), diffuse mild congestion (4/4) and multifocal moderate hemorrhages (1/4);<br>**Skeletal muscles**: multifocal moderate congestion and hemorrhages (4/4) and multifocal moderate to severe Zenker's necrosis (4/4);<br>**Heart**: diffuse moderate congestion (2/4) and multifocal moderate to severe hemorrhages (1/4).<br>**Liver**: diffuse mild steatosis (3/4) and diffuse mild to moderate congestion (4/4), multifocal moderate hemorrhages and necrosis (1/4);<br>**Spleen**: diffuse moderate congestion (3/4), associated with abundant melanomacrophages (1/4);<br>**Kidney**: diffuse moderate congestion (3/4) and hemorrhages in the hematopoietic tissue (2/4), eosinophilic amorphous material in tubular cells (3/4), vacuolar degeneration of the tubular epithelium (3/4), necrosis of the tubular epithelium (1/4);<br>**Brain**: diffuse moderate congestion (4/4) and multifocal moderate hemorrhages (2/4).	Illegal electro-fishing
		Carassius carassius (2)	**Skin**: diffuse mild hyperemia, mainly in the ventral region (2/2), and multifocal moderate hemorrhages (2/2);<br>**Gills**: diffuse moderate congestion (2/2);<br>**Heart**: hemopericardium (2/2);<br>**Stomach**: partially digested gastric content (2/2).	*1 animal examined*<br>**Skin**: multifocal moderate to severe hemorrhages;<br>**Gills**: diffuse moderate congestion;<br>**Skeletal muscles**: multifocal moderate Zenker's necrosis;<br>**Heart, liver, spleen, and kidney**: diffuse mild to moderate congestion**;**<br>**Brian**: diffuse moderate congestion.	
2	14	*Cyprinus carpio (5)*	**Eyes**: multifocal severe intraocular hemorrhages (3/5), exophthalmos (1/5);<br>**Gills**: diffuse moderate to severe hyperemia (5/5);<br>**Heart**: hemopericardium (4/5);<br>**Generalized** multifocal moderate to severe hemorrhages (5/5).	*1 animal examined*<br>**Eyes**: diffuse moderate vessels congestion and multifocal hemorrhages;<br>**Skeletal muscles**: multifocal moderate to severe Zenker's necrosis;<br>**Heart**: diffuse vessels congestion, petechiae in pericardium and pericardial fat;<br>**Swim bladder**: diffuse vessels congestion and multifocal hemorrhages;<br>**Brain**: diffuse vessels congestion and multifocal hemorrhages.	Illegal electro-fishing
		Mugil cephalus (2)	**Skin**: multifocal hemorrhages (2/2);<br>**Heart**: hemopericardium (2/2);<br>**Swim bladder**: diffuse vessels congestion (2/2).	*1 animal examined*<br>**Skeletal muscles**: multifocal moderate Zenker's necrosis;<br>**Heart**: diffuse moderate vessels congestion, presence of multifocal cysts;<br>**Liver**: diffuse steatosis and diffuse vessels congestion;<br>**Spleen**: diffuse vessels congestion.	
		Sander lucioperca (2)	**Skin**: multifocal moderate to severe hyperemia in head, ventral region and at the basis of fins (2/2);<br>**Heart**: hemopericardium (2/2).	*1 animal examined*<br>**Skeletal muscles**: multifocal moderate Zenker's necrosis;<br>**Heart**: diffuse moderate to severe vessels congestion;<br>**Liver, pancreas, spleen, and brain**: diffuse mild to moderate vessels congestion.	
		Silurus glanis (5)	**Skin**: diffuse hyperemia and disseminated hemorrhages (5/5);<br>**Stomach**: fresh aliment in gastric lumen (1/5).	*1 animal examined*<br>**Skeletal muscles**: multifocal moderate to severe Zenker's necrosis;<br>**Heart**: diffuse vessels congestion;<br>**Brain**: diffuse vessels congestion and focally extensive hemorrhages.	

3	35	*Abramis brama* (30)	**Skin**: multifocal moderate to severe hemorrhages (30/30);<br>**Eyes**: multifocal intraocular hemorrhages (30/30);<br>**Gills**: diffused moderate hyperemia (30/30)<br>**Heart**: hemopericardium (25-30/30)	*1 animal examined*<br>**Skin**: diffuse vessels congestion;<br>**Gills**: diffuse mild to moderate vessels congestion;<br>**Skeletal muscles**: multifocal moderate to severe Zenker's necrosis;<br>**Heart**: diffuse moderate vessels congestion;<br>**Liver**: mild diffuse vessels congestion;<br>**Brain**: diffuse vessels congestion.	Illegal electro-fishing
		Micropterus salmoides (1)	**Skin**: multifocal moderate hemorrhages;<br>**Eyes**: multifocal intraocular hemorrhages<br>**Gills**: diffuse moderate to severe hyperemia;<br>**Heart**: hemopericardium.	**Skin**: diffuse vessels congestion;<br>**Gills**: diffuse vessels congestion and multifocal moderate hemorrhages;<br>**Heart**: diffuse atrial congestion;<br>**Liver**: diffuse vessels congestion;<br>**Brain**: moderate diffuse vessels congestion.	
		Sander lucioperca (2)	**Skin**: multifocal moderate hemorrhages (2/2);<br>**Eyes**: multifocal intraocular hemorrhages (2/2);<br>**Gills**: diffuse moderate hyperemia (2/2);<br>**Heart**: hemopericardium (1/2).	*1 animal examined*<br>**Gills**: diffuse vessels congestion and multifocal severe hemorrhages, abundant presence of mucus and numerous *Trichodina* protozoans;<br>**Heart**: diffuse atrial congestion;<br>**Liver**: diffuse vessels congestion.	
		Silurus glanis (2)	**Skin**: multifocal moderate hemorrhages (2/2);<br>**Eyes**: multifocal moderate to severe intraocular hemorrhages (2/2);<br>**Gills**: diffuse moderate hyperemia (1/2);<br>**Heart**: hemopericardium (1/2);<br>**Stomach**: the gastric content was composed by dense yellow-greenish material (2/2).	*2 animals examined*<br>**Skin**: diffuse moderate vessels congestion (1/2);<br>**Skeletal muscles**: multifocal moderate Zenker's necrosis (1/2);<br>**Gills**: diffuse vessels congestion (1/2) and multifocal moderate telangiectasia (1/2);<br>**Heart**: diffuse vessels congestion (2/2);<br>**Liver**: diffuse moderate to severe hyperemia (2/2) and multifocal hemorrhages (2/2), and mild macrovacuolar steatosis (1/2);<br>**Spleen and kidney**: splenic pulp and kidney's interstitial hematopoietic tissue appeared rarefied (2/2), diffuse mild hyperemia (2/2) and multifocal hemorrhages (2/2);<br>**Pancreas**: diffuse vessels congestion (1/2) and multifocal moderate hemorrhages (1/2);<br>**Brain**: diffuse vessels congestion (2/2).	
4	4	*Cyprinus carpio* (4)	**Skin**: multifocal moderate hemorrhages (4/4);<br>**Eyes**: exophthalmos (4/4).	*4 animals examined*<br>**Sub-cutaneous tissue**: diffuse moderate congestion;<br>**Skeletal muscles**: diffuse mild to moderate congestion (4/4), diffuse moderate Zenker's necrosis (4/4) and multifocal moderate hemorrhages (1/4);<br>**Gills**: diffuse moderate congestion, multifocal hyperplasia, necrosis and epithelial sloughing (4/4);<br>**Heart**: diffuse vessels congestion (4/4).	Illegal electro-fishing
5	6	*Mugil cephalus* (1)	**Skin, periocular region, and skeletal muscles**: multifocal moderate to severe hemorrhages;<br>**Brain**: diffuse mild hyperemia.	**Eyes, skeletal muscles and gills**: diffuse moderate congestion.	Illegal electro-fishing
		Sander lucioperca (3)	**Skin**: multifocal moderate hemorrhages (1/3);<br>**Skeletal muscles**: multifocal moderate hemorrhages (1/3);<br>**Stomach**: partially digested gastric content (3/3).	*3 animals examined*<br>**Eyes, skeletal muscles, gills, and brain**: diffuse moderate to severe congestion (3/3).	
		Silurus glanis (2)	**Skin, sub-cutaneous tissue, and skeletal muscles**: diffuse mild to moderate hyperemia (in particular periocular, perioral, pectoral fins and left eye region) (2/2) and multifocal moderate to severe hemorrhages (2/2);<br>**Skin**: multifocal linear lesions (1/2);<br>**Liver**: multifocal moderate subcapsular petechiae (1/2)<br>**Stomach**: partially digested gastric content (1/2).	*2 animals examined*<br>**Skin**: multifocal severe epithelial erosion, petechiae and coagulative necrosis (2/2);<br>**Eyes**: diffuse moderate to severe vascular congestion (2/2);<br>**Skeletal muscles**: multifocal Zenker's necrosis (2/2), diffuse moderate to severe vascular congestion (2/2);<br>**Gills**: diffuse moderate to severe vascular congestion (2/2);<br>**Heart**: diffuse mild vessels congestion and multifocal myocardial petechiae (1/2);<br>**Brain**: diffuse moderate to severe vascular congestion (2/2).	

**6**	**5**	*Cyprinus carpio (5)*	**Skin**: multifocal moderate hemorrhages (5/5);<br>**Gills**: diffuse moderate hyperemia (5/5);<br>**Heart**: hemopericardium (5/5).	*5 animals examined*<br>**Skin**: diffuse moderate congestion e multifocal mild to moderate hemorrhages (2/5);<br>**Skeletal muscles**: diffuse moderate congestion (5/5), multifocal moderate hemorrhages (5/5) and Zenker's necrosis (5/5);<br>**Gills**: diffuse moderate congestion (5/5) multifocal epithelial necrosis (5/5), epithelial hyperplasia (2/5) and sloughing (5/5), multifocal telangiectasia (3/5); mild presence of metazoan parasites between lamellae (1/5);<br>**Heart**: diffuse moderate congestion (3/5) and multifocal myocardial petechiae (2/5);<br>**Liver**: diffuse moderate to severe congestion (5/5) and mild steatosis (4/5);<br>**Spleen**: diffuse mild congestion (3/5) and focal parasitic cyst (1/5);<br>**Kidney**: diffuse moderate congestion and multifocal hemorrhages in the hematopoietic tissue (2/5), diffuse mild to moderate congestion (5/5) and multifocal vacuolar degeneration of tubules epithelium (1/5), presence of Mixosporea spp. parasites in tubules lumen (1/5);<br>**Brain**: diffuse moderate congestion (5/5).	Illegal electro-fishing
**7**	**15**	*Cyprinus carpio (13)*	**Skin**: multifocal moderate to severe hemorrhages (13/13);<br>**Heart**: hemopericardium (11/13)	*3 animals examined*<br>**Skin**: diffuse mild to moderate congestion (2/3);<br>**Skeletal muscle**: multifocal Zenker's necrosis (2/3);<br>**Gills**: diffuse moderate congestion (2/3), multifocal epithelial hyperplasia (2/3), multifocal moderate hemorrhages (1/3);<br>**Heart**: diffuse mild congestion (3/3);<br>**Liver**: diffuse mild congestion (3/3);<br>**Spleen**: diffuse mild to moderate congestion (3/3);<br>**Kidney**: diffuse mild congestion and focal granulomatous inflammatory lesion (1/3);<br>**Brain**: diffuse moderate congestion (3/3).	Illegal elctro-fishing
		Sander lucioperca (2)	**Skin**: multifocal mild to moderate hemorrhages (2/2);<br>**Heart**: hemopericardium (2/2)	*1 animal examined*<br>**Skin**: diffuse moderate congestion;<br>**Skeletal muscle**: multifocal Zenker's necrosis;<br>**Gills**: diffuse moderate congestion;<br>**Heart**: diffuse mild congestion<br>**Liver**: diffuse moderate congestion and mild steatosis;<br>**Spleen and kidney**: diffuse mild congestion;<br>Brain: diffuse mild congestion.	
**8**	**39**	*Cyprinus carpio (37)*	**Skin**: multifocal moderate hemorrhages (39/39);<br>**Eyes**: exophthalmos (39/39);<br>**Gills**: diffuse severe hyperemia (39/39);<br>**Heart**: hemopericardium (39/39).	*2 animals examined*<br>**Skin**: diffuse moderate to severe congestion (2/2);<br>**Skeletal muscles**: multifocal moderate Zenker's necrosis (1/2);<br>**Heart**: diffuse mild to moderate congestion (2/2);<br>**Liver**: diffuse mild to moderate congestion (2/2);<br>**Brain**: diffuse mild congestion (2/2).	Illegal electro-fishing
		Silurus glanis (2)		*1 animal examined*<br>**Skin**: diffuse moderate congestion;<br>**Skeletal muscles**: diffuse mild to moderate congestion and Zenker's necrosis.	
**9**	**3**	*Hypophthalmichthys molitrix (3)*	**Skin, sub-cutaneous tissue, fins, and anal mucosa**: diffuse moderate to severe hemorrhages (9/9);<br>**Eyes**: exophthalmos (9/9);<br>**Gills**: diffuse moderate to severe congestion (9/9);<br>**Heart**: hemopericardium (9/9).	*1 animal examined*<br>**Skin**: diffuse moderate congestion<br>**Skeletal muscles**: multifocal moderate Zenker's necrosis;<br>**Gills**: diffuse severe congestion;<br>**Heart**: diffuse moderate congestion;<br>**Liver**: diffuse severe post-mortem degeneration;<br>**Kidney**: diffuse moderate congestion and diffuse hyperemia of hematopoietic tissue;<br>**Brain**: diffuse moderate congestion.	Illegal electro-fishing
